# Transcriptional analysis of the expression, prognostic value and immune infiltration activities of the COMMD protein family in hepatocellular carcinoma

**DOI:** 10.1186/s12885-021-08699-3

**Published:** 2021-09-07

**Authors:** Xiaobo Wang, Shujiao He, Xin Zheng, Shanzhou Huang, Honghui Chen, Huadi Chen, Weixin Luo, Zhiyong Guo, Xiaoshun He, Qiang Zhao

**Affiliations:** 1 Organ Transplant Center, the First Affiliated Hospital, Sun Yat-Sen University, NO.58 Zhongshan Road, Guangzhou, 510080 China; 2 Guangdong Provincial Key Laboratory of Organ Donation and Transplant Immunology, Guangzhou, 510080 China; 3 Guangdong Provincial International Cooperation Base of Science and Technology, Guangzhou, 510080 China; 4 Department of Orthopaedics, the Affiliated Hospital of Xuzhou Medical University, Xuzhou, China; 5 Department of General Surgery, Guangdong Provincial People’s Hospital, Guangdong Academy of Medical Sciences, School of Medicine, South China University of Technology, Guangzhou, China

**Keywords:** COMMD protein family, Hepatocellular carcinoma, mRNA, Transcription, Prognosis, Immune infiltration

## Abstract

**Background:**

The copper metabolism MURR1 domain (COMMD) protein family involved in tumor development and progression in several types of human cancer, but little is known about the function of COMMD proteins in hepatocellular carcinoma (HCC).

**Methods:**

The ONCOMINE and the UALCAN databases were used to evaluate the expression of COMMD1–10 in HCC and the association of this family with individual cancer stage and tumor grade. Kaplan-Meier (K-M) Plotter and Cox analysis hint the prognostic value of COMMDs. A network comprising 50 most similar genes and COMMD1–10 was constructed with the STRING database. Gene set enrichment analysis (GSEA) was performed using LinkedOmics database. The correlations between COMMD expression and the presence of immune infiltrating cells were also analyzed by the tumor immune estimation resource (TIMER) database. GSE14520 dataset and 80 HCC patients were used to validated the expression and survival value of COMMD3. Human HCC cell lines were also used for validating the function of COMMD3.

**Results:**

The expression of all COMMD family members showed higher expression in HCC tissues than that in normal tissues, and is associated with clinical cancer stage and pathological tumor grade. In HCC patients, the transcriptional levels of COMMD1/4 are positively correlated with overall survival (OS), while those of COMMD2/3/7/8/9 are negatively correlated with OS. Multivariate analysis indicated that a high level of COMMD3 mRNA is an independent prognostic factor for shorter OS in HCC patients. However, the subset of patients with grade 3 HCC, K-M survival curves revealed that high COMMD3/5/7/8/9 expression and low COMMD4/10 expression were associated with shorter OS. In addition, the expression of COMMD2/3/10 was associated with tumor-induced immune response activation and immune infiltration in HCC. The expression of COMMD3 from GSE14520 dataset and 80 patients are both higher in tumor than that in normal tissue, and a higher level of COMMD3 mRNA is associated with shorter OS. Knockdown of COMMD3 inhibits human HCC cell lines proliferation in vitro.

**Conclusions:**

Our study indicates that COMMD3 is an independent prognostic biomarker for the survival of HCC patients. COMMD3 supports the proliferation of HCC cells and contributes to the poor OS in HCC patients.

**Supplementary Information:**

The online version contains supplementary material available at 10.1186/s12885-021-08699-3.

## Background

Globally, primary liver cancer is the sixth most prevalent malignant tumor and the fourth leading cause of cancer-related death [1], and approximately 85–90% of primary liver cancers are hepatocellular carcinoma (HCC) [2]. Various treatment strategies, such as hepatectomy, transplantation, ablation, and interventional therapy, have improved survival benefits for HCC patients [3], but the high recurrence rate has led to an unsatisfactory prognosis and poor overall survival (OS) for these patients [4]. Although there are clear data regarding the relationship between the molecular pathogenesis of HCC and tumorigenesis, progression and clinical outcome, the specific molecular characteristics of HCC that drive this association remain limited [5]. Therefore, it is critical to clarify the molecular mechanism(s) involved in HCC and identify accurate prognostic biomarkers to effectively manage and treat HCC patients.

The copper metabolism MURR1 domain (COMMD) protein family consists of 10 family members, all of which have a highly structurally conserved C-terminal motif [6]. Increasing evidences show that COMMD proteins play important roles in tumorigenesis, progression, invasion, and metastasis. COMMD1, the first characterized COMMD protein, participates in multiple processes, such as copper metabolism, sodium excretion, and inflammatory responses [6–8]. COMMD1 causes tumor apoptosis in lung cancer by suppressing SOD1 expression [9], and in neuroblastoma, elevated nuclear expression of COMMD1 inhibits cyclin D1 expression, G1/S transition, and tumor cell proliferation [10]. It has also been found that COMMD1 associated with better OS. For example, overexpression of COMMD1 in colorectal cancer, glioblastoma, and melanoma could suppress cancer cells invasion and metastasis by directly inhibiting HIF-mediated gene expression [11]. Despite these data, only COMMD7 has been investigated in HCC. It has been shown to promote the migration and invasion of HCC cells by upregulating C-X-C motif chemokine 10 (CXCL10) expression [12], while COMMD7 knockdown in HCC inhibited cell proliferation, migration, and invasion via suppressing NF-κB [13]. However, little is known about the function of all COMMD family members in HCC.

In this study, the Kaplan-Meier (K-M) Plotter database in combination with univariate Cox analysis and multivariate Cox analysis was used to assess HCC-related mRNA sequencing data from The Cancer Genome Atlas (TCGA) database in order to identify the prognostic and predictive values of COMMD proteins in HCC patients. A comprehensive evaluation of the possible antitumor mechanisms of COMMD proteins and the correlation of these proteins with the immune microenvironment phenotype was also performed. Finally, the expression and survival value of COMMD3 were validated in GSE14520 dataset and 80 patients, and the COMMD3 functional studies were performed in Human HCC cell lines.

## Methods

### ONCOMINE database

The ONCOMINE database (www.oncomine.org) is an integrated online cancer microarray database containing DNA and RNA sequences that is designed to facilitate investigations using genome-wide expression analyses [14]. In this study, a *p-value* of 0.01, fold change of 1.5, gene rank of 10%, and data type of mRNA were set as the inclusion criteria. Transcriptional expression data of COMMD1–10 in cancer and normal tissues were downloaded from the ONCOMINE database. The differences in expression were compared by Student’s t-test.

### UALCAN

UALCAN (http://ualcan.path.uab.edu) is an interactive web portal that can be analyzed based on RNA-seq and clinical data of 31 cancer types for in-depth analysis of TCGA gene expression data [15]. One feature is its ability to identify relative changes in the transcriptional expression of target genes between tumor and normal samples and associations between transcriptional expression and relative clinicopathological parameters. In this study, UALCAN was used to analyze the mRNA expression of the 10 COMMD family members in HCC tissues and the association of these proteins with clinicopathological parameters. Differences in transcriptional expression were compared by Student’s t-test, and *p* < 0.01 was considered statistically significant.

### Kaplan-Meier plotter

K-M Plotter (http://kmplot.com/analysis/) is an integrated platform that can correlate gene expression with survival for multiple cancers, including liver, gastric, and breast [16–18]. This study analyzed the prognostic value of COMMD proteins in HCC by stratifying cancer patients into high and low expression groups based on the median mRNA expression values for each gene; the stratifications were validated using K-M survival curves. Information about the number-at-risk cases, median values of the mRNA expression level, HRs, 95% CIs and *p*-values can be found on the K-M Plotter website. A statistically significant difference was considered when a *p-value* < 0.05.

### The cancer genome atlas database

The TCGA database is a comprehensive and coordinated project comprising sequencing and clinical data of more than 30 human cancers [19]. In our analysis, the clinicopathological parameters of 377 HCC patients and the COMMD mRNA expression data of 421 HCC patients were downloaded. Samples lacking follow-up data or COMMD expression were excluded, and the COMMD expression data from 311 HCC patients were subjected to univariate and multivariate Cox analyses.

### cBioPortal

cBioPortal (www.cbioportal.org) is a web resource that allows for the exploration, visualization, and analysis of multidimensional cancer genomics data [20]. We analyzed the genomic profiles of 10 members of the COMMD family, including mutations from GISTIC and mRNA expression z-scores (RNASeq V2 RSEM) with a z-score threshold ±1.8. Genetic mutations in COMMD proteins and their association with OS, disease-free survival (DFS), and progression-free survival (PFS) of HCC patients are presented as K-M plots. A log-rank test was performed to determine the significant difference between the survival curves, with a *p-value* < 0.05 indicating a statistically significant difference.

### Immune scores

Estimation of STromal and Immune cells in MAlignant Tumor Tissues using Expression data (ESTIMATE) is an algorithm that uses gene expression signatures to predict the ratios of stromal and immune cells in tumor samples. Immune scores of HCC patients from the TCGA database can be downloaded from the website https://bioinformatics.mdanderson.org/estimate [21]. We divided the HCC cases into two groups based on the median value of the immune score, with the high-score group comprising individuals with higher immune scores and the low-score group comprising individuals with lower immune scores.

Tumor immune estimation resource (TIMER) is a web resource for the comprehensive analysis of tumor-infiltrating immune cells (https://cistrome.shinyapps.io/timer/) [22]. The composition of the immune infiltrate (e.g., B cells, CD4+ T cells, CD8+ T cells, neutrophils, macrophages, natural killer cells (NKs) and dendritic cells (DCs)) can be statistically estimated from gene expression profiles; these estimations are validated using pathological assessments. In addition, the “correlation” module can be used to evaluate the expression of a pair of genes in a specific cancer type and analyze the Spearman correlation and statistical significance. Therefore, we used this module to explore the correlations between COMMD protein expression and gene markers of immune infiltrating cells in HCC.

### GEPIA2 and STRING databases

The Gene Expression Profiling Interactive Analysis 2 (GEPIA) web server is a resource for gene expression analysis based on tumor and normal samples from the TCGA and GTEx databases [23]. Here, the module “Similar Gene Detection” (http://gepia2.cancer-pku.cn/#similar) was used to identify the five most similar genes for each COMMD family member. The STRING database functions as a repository for the collection, scoring, and integration of all publicly available sources of information relating to protein-protein interactions and aims to complement these data with computational predictions [24]. A network composed of COMMD1–10 and their 50 similar neighboring genes was constructed using a protein-protein interaction module.

### Gene set enrichment analysis (GSEA)

The LinkedOmics database (http://www.linkedomics.org) contains multiomics and clinical data for 32 cancer types encompassing 11,158 patients from the TCGA database [25]. Here, the LinkedOmics database was used for GSEA to identify the biological processes enriched for the COMMD proteins. Gene sets with nominal *p-value* < 0.05 and a false discovery rate (FDR) < 0.25 were considered statistically significant.

### GEO datasets

GSE14520 dataset was obtained from the Gene Expression Omnibus (GEO, https://www.ncbi.nlm.nih.gov/geo/) for the validation studies.

### RNA extraction and qRT-PCR analysis

RNA from fresh frozen normal liver and tumor tissues obtained from 80 HCC patients treated at our hospital between December 2016 and December 2019 was extracted with TRIzol reagent (Thermo Fisher, USA). Reverse transcription was performed with oligo dT and RevertAid Reverse Transcriptase (Thermo Fisher, USA), and the resulting cDNAs were subjected to quantitative PCR with SuperReal PreMix SYBR Green (TIANGEN, China) in an Applied Biosystems 7500 Fast Real-Time PCR System (Life Technologies, USA). The amplification primers were purchased from RIBO, China. The expression levels were normalized to those of ACTB1. Amplification primers were as follows:

- COMMD3 Forward primer 5–3: TTGACAGAGAGCGAATAGAACTG
- COMMD3 Reverse primer 5–3: TGAGGGAGAGATCTGCCTATAC

### Cell culture and transient transfection

Human HCC cell lines, HepG2 and Hep3B were obtained from ScienCell Research Laboratories. They were cultured in DMEM containing 10% fetal bovine serum (Gibco, Carlsbad, CA, USA). Both cell lines were maintained in a humidified incubator with 37 °C, 5% CO2. Lipofectamine RNAiMAX (ThermoFisher, USA) was used to transfect Negative Control (NC) and COMMD3 siRNAs (Ribobio, China) into HCC cells as the manufacturer’s protocol suggested. Target sequences for siRNAs were TGGTGACCTTAAGTGTACA (COMMD3 siR1), GCAGATCTCTCCCTCATAT (COMMD3 siR2) and CGCTTGGAATATCAGATAA (COMMD3 siR3).

### Cell proliferation assay

After exploring the transfected efficiency of COMMD3 siRNAs by Western Blotting (Supplementary Fig. 1), cell proliferation analysis was performed. Cells were seeded into 48-well culture plates (1 × 10^4^ cells/well). After 12 h cultivated, siRNAs were transfected. Thirty microliter CCK-8 solution (Dojindo, Kumamoto, Japan) was added to each well at different time points and incubated for 1.5 h. Samples’ optical density (OD) was measured immediately at 450 nm.

The Cell-Light 5-ethynyl-2-deoxyuridine (EdU) Apollo567 In Vitro Kit (Ribobio, China) was also used to assess cell proliferation. Cells transfected with miRNA mimics for 24 h were seeded into 6-well plates (2 × 10^5^ cells/well). After 12 h cultivated, the cells were transfected for 24 h and were cultivated for 40 h. Then the cells were incubated in EdU working solution for 2 h, fixed with 4% paraformaldehyde, permeabilized, washed, and stained with 1x Apollo solution and 1x Hoechst33342 solution according to the manufacturer’s instructions. Results were analyzed from microphotographs taken using a fluorescence microscope.

### Statistical methods

Cox regression analysis in R 3.6.1 software was used to evaluate the association of mRNA expression of the COMMD genes with patient survival. The effect of clinical parameters and mRNA expression of the COMMD genes on the survival of HCC patients was evaluated by univariate Cox regression; genes with *p-value* ≤ 0.05 in the univariate analysis were then included in the subsequent multivariate analysis. An unpaired t-test was used to compare the expression of COMMD proteins between the high and low immune score groups. GraphPad Prism (v. 7.0) was used for statistical analysis and figure creation. The different expression of COMMD3 between normal and tumor tissue in GSE14520 was compared using Limma packages, while the difference in our patients was compared by paired t-test. An unpaired t-test was used for cell proliferation assays (CCK8 and EdU assay). GraphPad Prism (v. 7.0) was used for statistical analysis and figure creation. *p-value* < 0.05 was considered statistically significant.

## Results

### Transcriptional levels of the COMMD genes are increased in patients with HCC

**Fig. 1:**
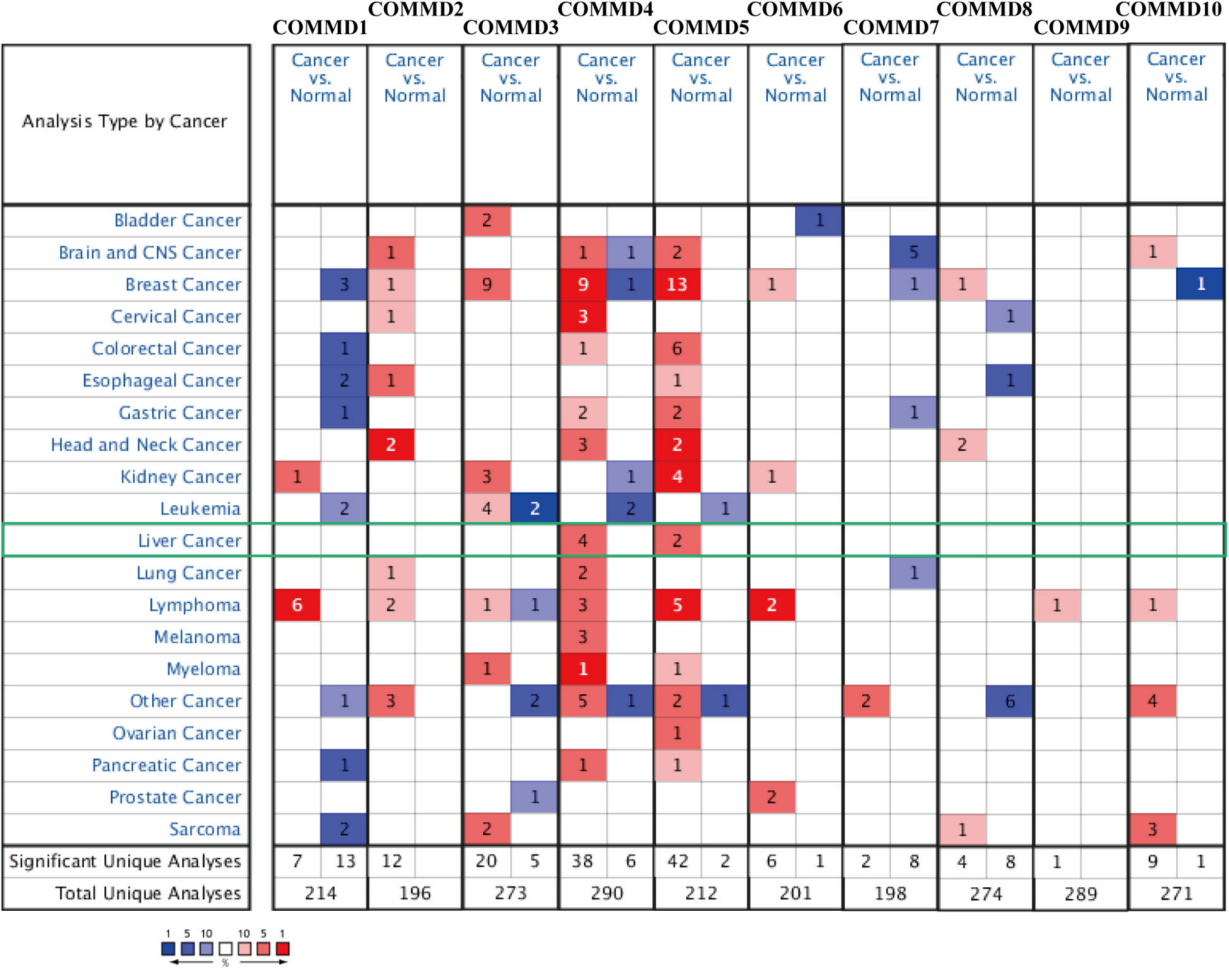
Differences in transcriptional expression in different cancers from ONCOMINE database. The cutoff values for the inclusion criteria were as follows: *p-value*, 0.01; fold change, 1.5; gene rank, 10%; and data type, mRNA

Ten COMMD family members have been identified in mammals. We compared the transcriptional levels of COMMD1–10 between HCC and normal tissues using the ONCOMINE database and UALCAN. The mRNA expression of COMMD4 in HCC patients was significantly upregulated in the four datasets (Fig.1). In the Chen liver dataset, COMMD4 was higher in HCC tissues than in normal tissues (fold change 1.669, *p* = 3.22E-15) (Table 1) [26]. Wurmbach et al. observed a 1.663-fold increase (*p* = 5.37E-7) in COMMD4 mRNA expression in HCC tissues compared to normal tissues [27]. COMMD4 mRNA expression was reported to be 1.576-fold higher (*p* = 2.36E-43) and 1.793-fold higher (*p* = 2.36E-43) in the first and second Roessler liver datasets [28]. In addition, Chen et al. and Wurmbach et al. reported that COMMD5 expression was 1.505-fold and 1.642-fold higher, respectively, in HCC samples than in normal samples (Table 1) [27, 28].

**Table 1:** Significant changes of COMMDs expression in transcriptional level between HCC and normal tissues (ONCOMINE)

	Types of HCC VS. Liver	Fold Change	*p-value*	t-test	Ref
COMMD4	HCC	1.669	3.22E-15	8.524	Chen Liver [26]
	HCC	1.663	5.37E-7	6.679	Wurmbach Liver [27]
	HCC	1.576	2.58E-7	6.133	Roessler Liver [28]
	HCC	1.793	2.36E-43	15.609	Roessler Liver 2 [28]
COMMD5	HCC	1.505	1.64E-10	6.690	Chen Liver [26]
	HCC	1.642	3.38E-5	4.564	Wurmbach Liver [27]

*HCC* Hepatocellular carcinoma, *COMMDs* Copper metabolism MURR1 domain (COMMD) protein family.

**Fig. 2:**
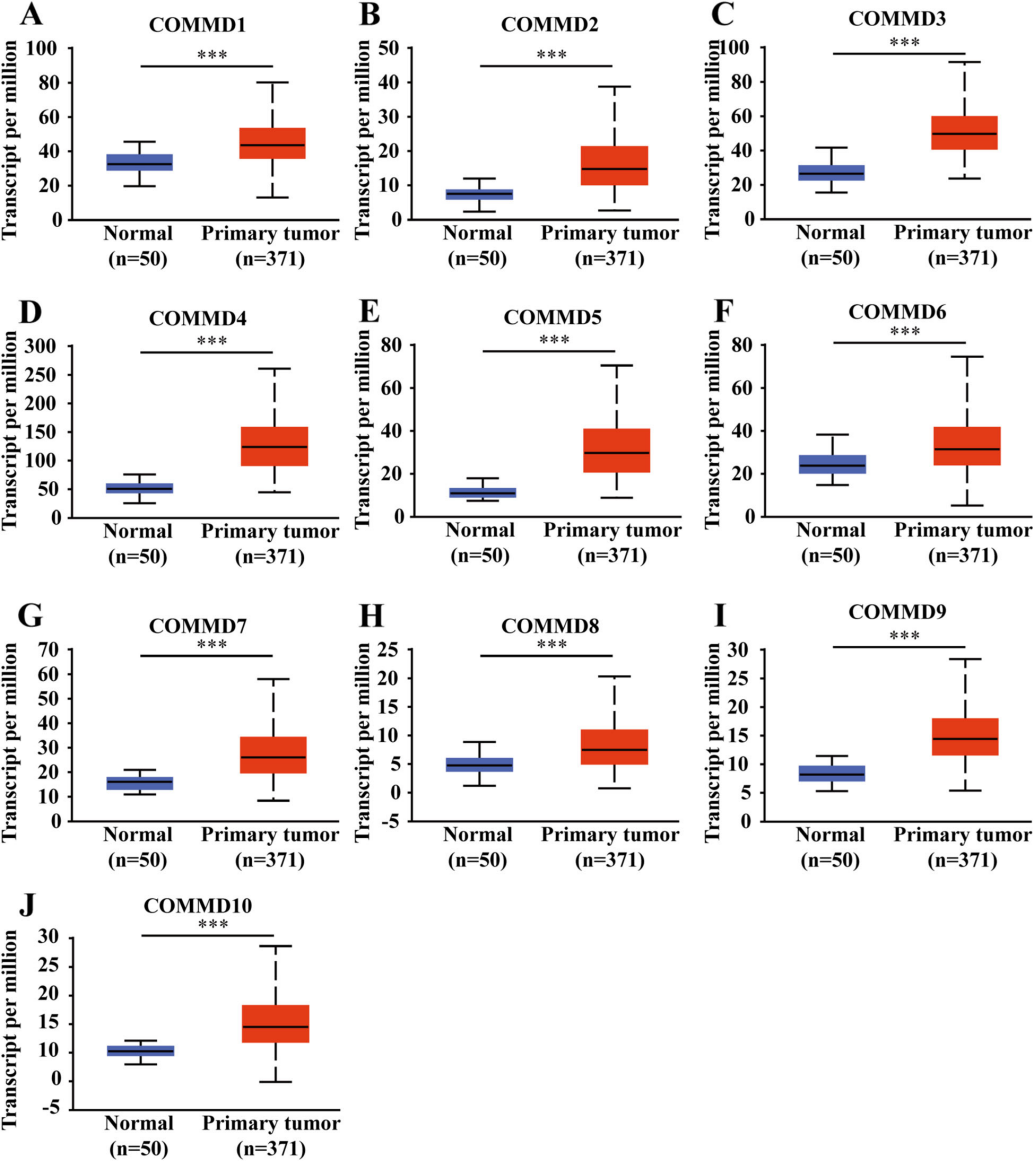
Transcriptional expression of COMMD genes in HCC tissues and normal tissues. **A-J** mRNA expression of the10 COMMD family members was higher in primary HCC tissues than in normal samples (UALCAN). *** *p* < 0.001

To validate these results, we explored the mRNA expression profile of COMMD1–10 in UALCAN, which has different source material and data than the ONCOMINE database. All 10 COMMD family members were found to be significantly overexpressed in primary HCC tissues compared to normal samples (Fig. 2A-J, *p* < 0.05).

### Relationship between the mRNA expression level of COMMD1–10 and clinicopathological parameters in HCC patients

**Fig. 3:**
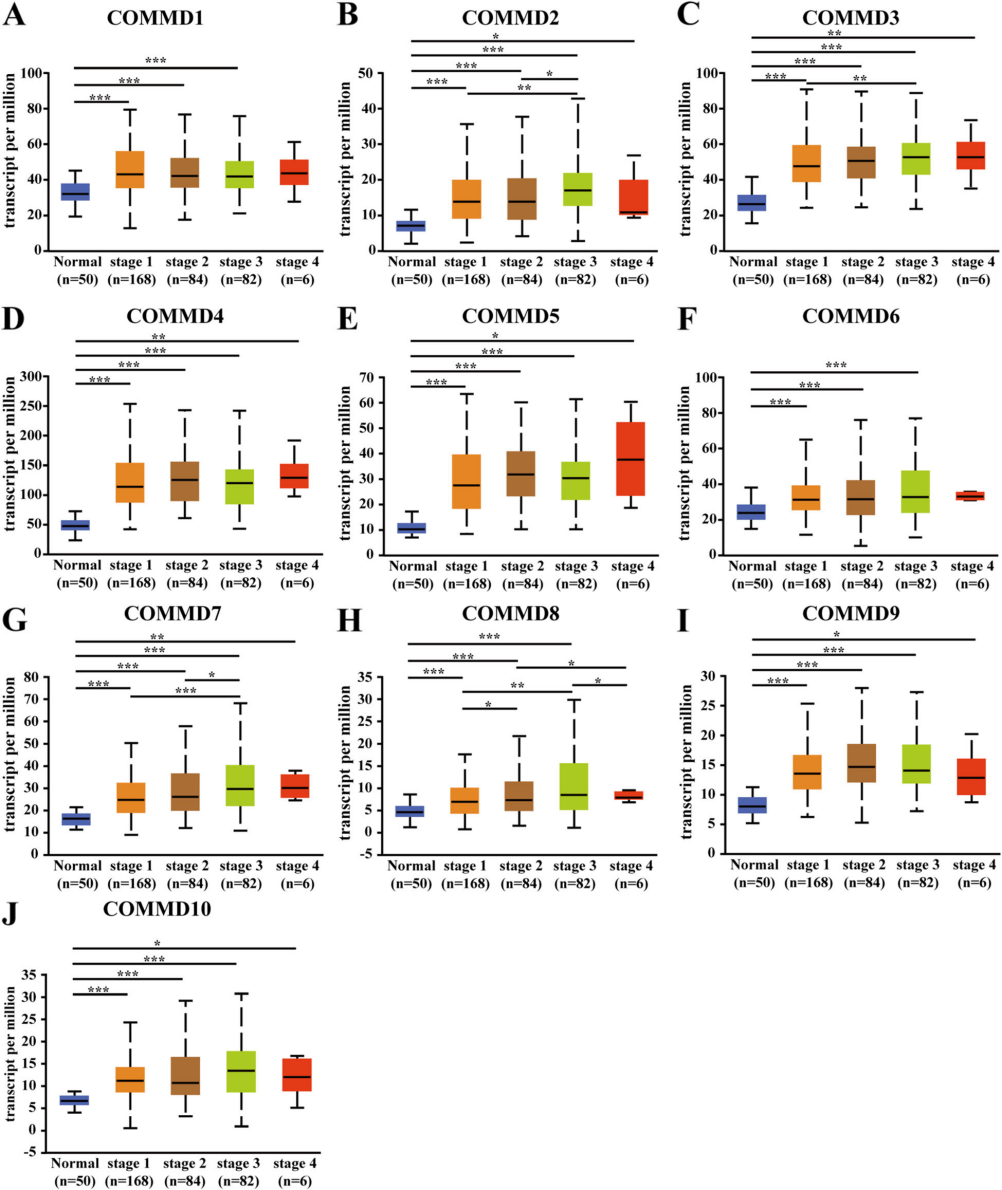
Relationship between the mRNA expression of COMMD genes and individual cancer stage of HCC patients. **A-J** The mRNA expression of the 10 COMMD genes was correlated with the patients’ individual cancer stage. * *p* < 0.05, ** *p* < 0.01, *** *p* < 0.001

**Fig. 4:**
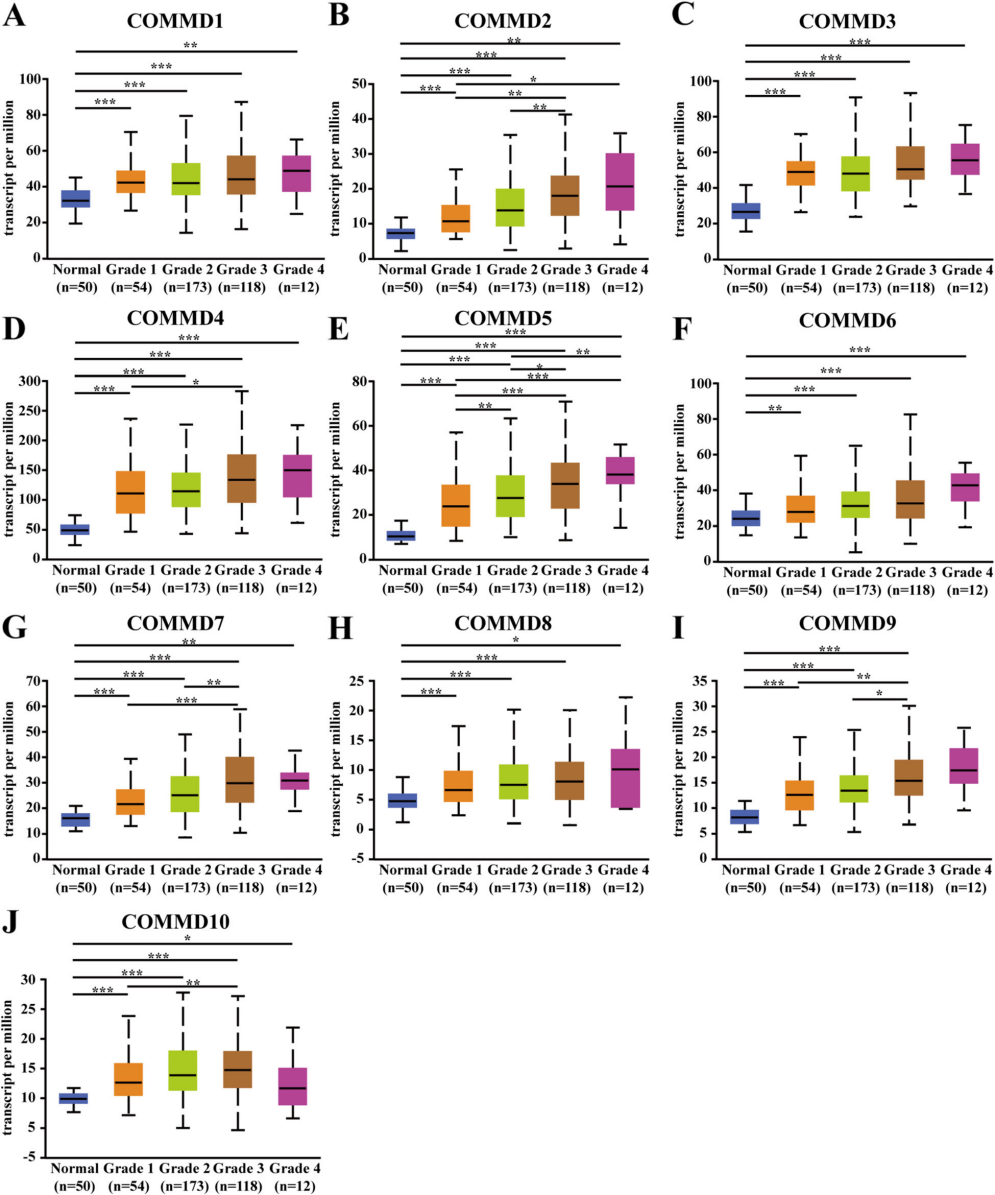
Relationship between the mRNA expression of COMMD genes and tumor grade of HCC patients. **A-J** The mRNA expression of 10 COMMD genes was correlated with patient tumor grade. * *p* < 0.05, ** *p* < 0.01, *** *p* < 0.001

Next, we analyzed the relationship between the mRNA expression of COMMD1–10 and clinicopathological parameters of HCC patients (individual cancer stage and tumor grade) through UALCAN, including patients’ individual cancer stage and tumor grade. The mRNA expression levels of COMMD1–10 were correlated with individual cancer stage: patients with a more advanced cancer stage tended to express higher mRNA levels of COMMD proteins (Fig. 3A-J). The highest levels of COMMD1/3/4/5/6/7 mRNA expression were observed in patients with stage 4 disease (Fig. 3A, C, D-G), while the highest levels of COMMD2/8/10 mRNA expression were in patients with stage 3 disease (Fig. 3B, H, J). Interestingly, the highest levels of COMMD9 mRNA expression were stage 2 patients (Fig. 3I); the primary reason for the peaked mRNA expression in patients with stage 2/3 disease seemed to be related to the small sample size of stage 4 patients. Similarly, the mRNA expression levels of COMMD1–10 were significantly related to tumor grade. The mRNA levels of COMMD tended to be higher as the tumor grade increased. The highest mRNA expression levels of COMMD1/2/3/4/5/6/7/8/9 were found in grade 4 tumors (Fig. 4A-I), while the highest mRNA expression of COMMD10 was found in grade 3 tumors (Fig. 4J). In summary, these results suggested that the mRNA expression levels of COMMD1–10 were significantly associated with clinicopathological parameters in HCC patients.

### Prognostic value of mRNA expression of COMMD proteins in HCC patients

**Fig. 5:**
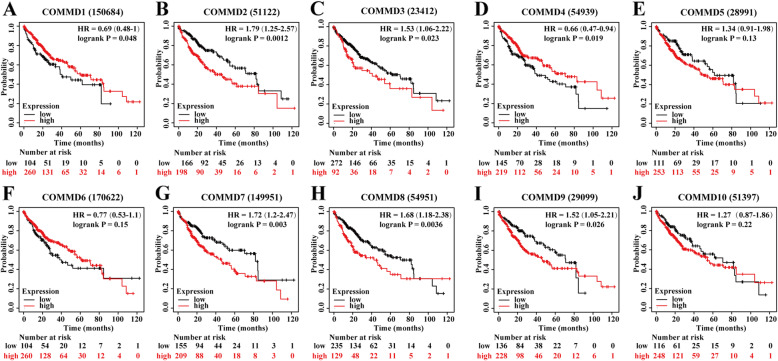
Prognostic value of the mRNA expression of COMMD genes in HCC patients (K-M Plotter). **A-J** K-M OS curves comparing all HCC patients stratified by high and low expression of COMMD1–10. *p < 0.05

**Fig. 6:**
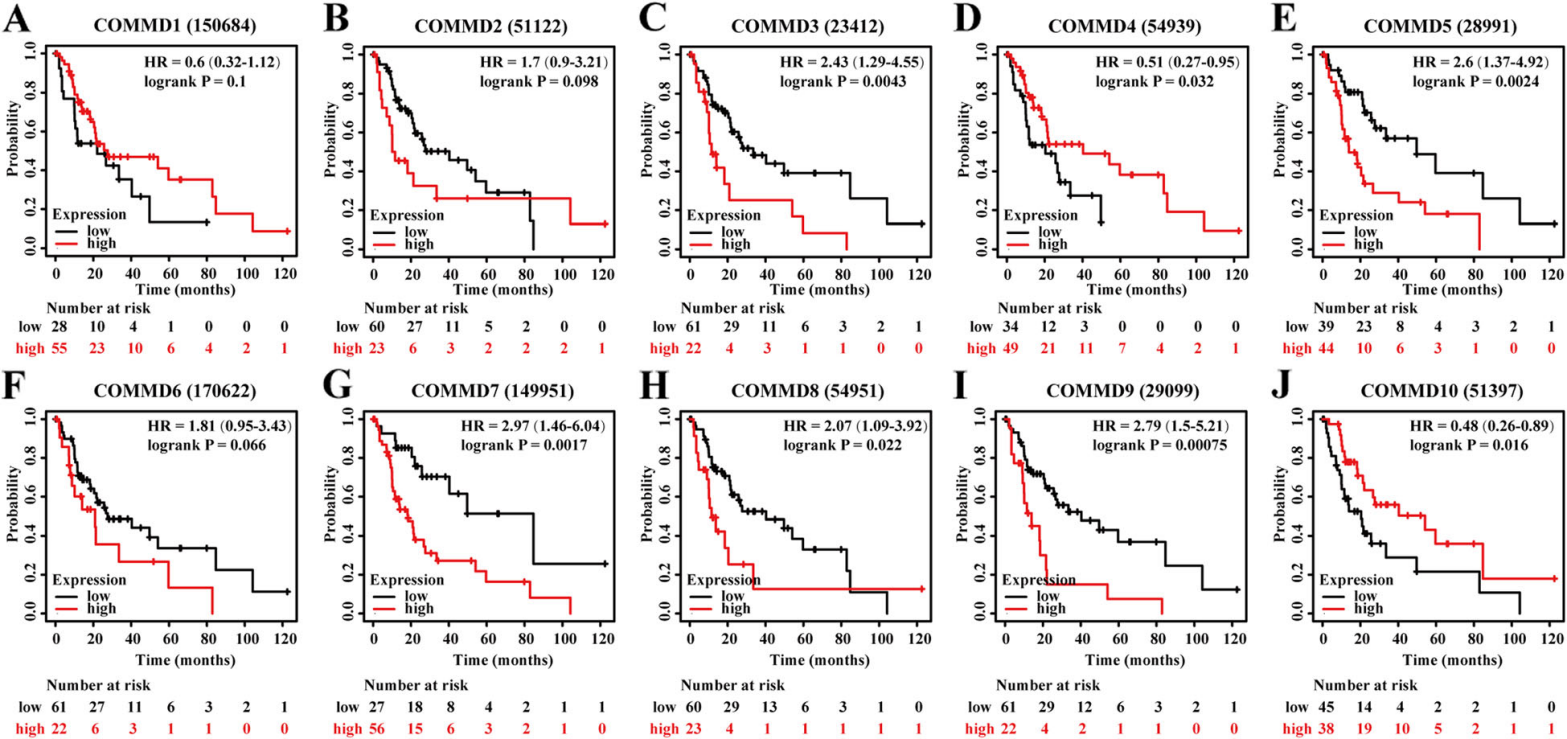
Prognostic value of the mRNA expression of COMMD proteins in patients with grade III HCC (K-M Plotter). **A-J** K-M OS curves comparing high and low expression of COMMD1–10 in patients with grade III HCC. *p* < 0.05

The Kaplan-Meier Plotter database was used to explore the correlation between the mRNA levels of COMMD1–10 and the survival of HCC patients. The K-M curves and log-rank test analysis revealed that the mRNA levels of most COMMD family members were significantly associated with OS (*p* < 0.05) (Fig. 5A-J) in HCC patients. Higher mRNA expression of COMMD2/3/7/8/9 was related to shorter OS (Fig. 5 BCG-I), while higher levels of COMMD1 and COMMD4 were related to longer OS (Fig. 5 AD). Since higher mRNA levels of COMMD proteins were found in patients with a more advanced cancer grade and the cohort of individuals with grade 4 disease was small, we further explored the correlation between the mRNA levels of COMMD1–10 and the survival of HCC patients with grade 3 disease. As shown in Fig. 6A-J, higher mRNA levels of COMMD3/5/7/8/9 were associated with poor OS (Fig. 6CEG-I) while higher mRNA levels of COMMD4/10 were associated with a more favorable OS (Fig. 6DJ). These results indicated that the mRNA expression of COMMD1/2/3/4/7/8/9 was markedly associated with prognosis in liver cancer patients and that the mRNA expression levels of COMMD3/4/5/7/8/9/10 were significantly related to prognosis in patients with grade 3 HCC. Based on these observations, the COMMD genes may be exploited as useful biomarkers for predicting HCC patient survival.

### Independent prognostic value of mRNA expression of COMMD proteins in terms of OS in HCC patients

Data associated with 344 HCC patients with complete mRNA expression and clinical information downloaded from the TCGA database were used for Cox survival regression analysis; clinical characteristics are shown in Supplementary Table 1. Among all patients with HCC, univariate analysis showed that advanced age (HR = 1.02, 95% CI: 1.00–1.04, and *p* = 0.024), high mRNA expression of COMMD3 (HR = 2.37, 95% CI: 1.48–3.8, and *p* < 0.001) and high mRNA expression of COMMD5 (HR = 1.65, 95% CI: 1.05–2.59, and *p* = 0.029) were related to significantly shorter OS (Table 2). Multivariate analysis revealed that only high mRNA expression of COMMD3 (HR = 2.16, 95% CI: 1.33–3.48, and *p* = 0.002) was independently associated with significantly shorter OS among all HCC patients (Table 2) indicating that COMMD3 is an independent prognostic factor for OS in all HCC patients.

**Table 2:** Univariate and multivariate analysis of overall survival in 344 HCC patients

Variables	Univariate analysis			Multivariate analysis		
	Hazard Ratio	CI 95%	P	Hazard Ratio	CI 95%	P
Age (≤60 / > 60)	1.02	1–1.04	0.024*	1.02	1–1.04	0.05
Gender (M / F)	1.43	0.9–2.26	0.129			
Grade (I + II / III + IV)	1.16	0.92–1.46	0.218			
AJCC stage (I + II / III + IV)	1.23	0.96–1.58	0.102			
COMMD1 (high / low)	1.14	0.72–1.79	0.579			
COMMD2 (high / low)	0.97	0.61–1.55	0.903			
COMMD3 (high / low)	2.37	1.48–3.8	0.000*	2.16	1.33–3.48	0.002*
COMMD4 (high / low)	1.07	0.67–1.69	0.788			
COMMD5 (high / low)	1.65	1.05–2.59	0.029*	1.33	0.83–2.12	0.233
COMMD6 (high / low)	1.23	0.76–1.99	0.398			
COMMD7 (high / low)	0.82	0.5–1.34	0.437			
COMMD8 (high / low)	1.5	0.94–2.4	0.091			
COMMD9 (high / low)	1.44	0.89–2.31	0.137			
COMMD10 (high / low)	1.06	0.67–1.68	0.794			

*HCC* Hepatocellular carcinoma, *CI* Confidence interval. * *p* < 0.05.

In 124 patients with grade 3 HCC (clinical characteristics are listed in Supplementary Table 2), univariate analysis showed that high mRNA expression of COMMD3 (HR = 2.82, 95% CI: 1.40–5.70, and *p* = 0.004) was related to shorter OS (Table 3). These results demonstrated that COMMD3 is an independent prognostic factor for OS in patients with grade 3 or 4 HCC.

**Table 3:** Univariate and multivariate of overall survival in 130 patients with HCC of grade III and IV

Variables	Univariate analysis			Multivariate analysis		
	Hazard Ratio	CI 95%	P	Hazard Ratio	CI 95%	P
Age (≤60 / > 60)	1.02	0.99–1.05	0.15			
Gender (M / F)	0.99	0.49–2.02	0.979			
AJCC stage (I + II / III + IV)	1.3	0.88–1.91	0.182			
COMMD1 (high / low)	1.33	0.67–2.63	0.416			
COMMD2 (high / low)	1.28	0.64–2.58	0.485			
COMMD3 (high / low)	2.82	1.4–5.7	0.004*	2.82	1.4–5.7	0.004*
COMMD4 (high / low)	0.8	0.4–1.61	0.534			
COMMD5 (high / low)	1.95	0.98–3.9	0.057			
COMMD6 (high / low)	1.21	0.61–2.4	0.586			
COMMD7 (high / low)	0.76	0.37–1.58	0.466			
COMMD8 (high / low)	1.5	0.75–2.98	0.254			
COMMD9 (high / low)	1.32	0.63–2.78	0.467			
COMMD10 (high / low)	1.12	0.55–2.28	0.756			

*HCC* Hepatocellular carcinoma, *CI* Confidence interval. * *p* < 0.05.

### Relationship between COMMD expression and immune infiltration in HCC

**Fig. 7:**
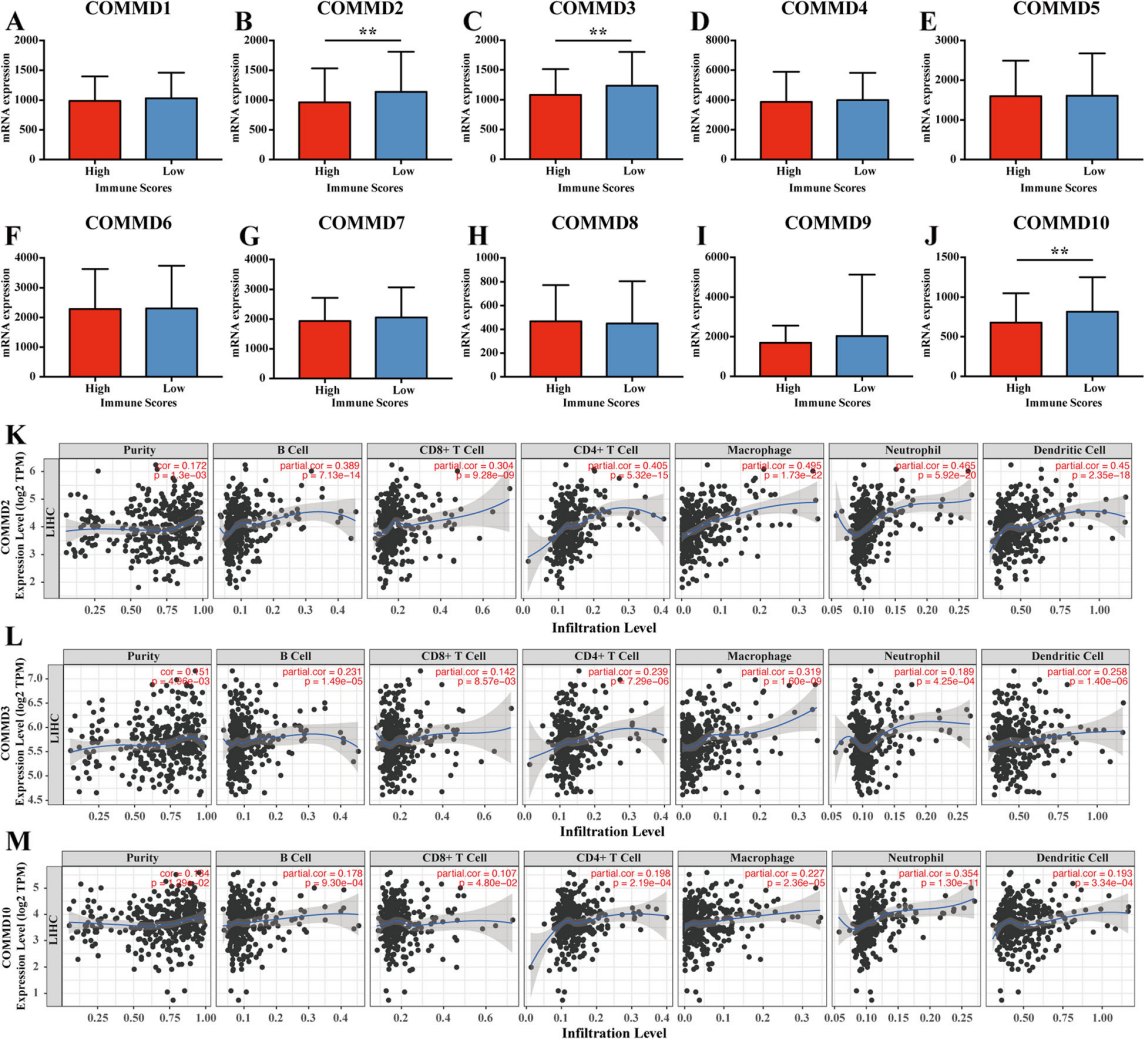
Correlation of COMMD expression with the level of immune infiltration in HCC. **A-J** Comparison of COMMD expression between the high and low immune score groups comprising HCC patients. **K-M** COMMD2/3/10 were related to tumor purity and immune infiltration levels of HCC by TIMER analysis. ** *p* < 0.01

The immune scores of patient samples from the TCGA database were calculated by the ESTIMATE algorithm in order to predict the presence of infiltrating immune cells in tumor tissues. According to the immune score ranks, we divided the HCC cases into high- and low-score groups by the median value (Fig. 7A-J). The results showed that COMMD2/3/10 expression was significantly lower in the high-score group, which indicates that COMMD2/3/10 might be involved in immune infiltration in HCC (*p* < 0.05) (Fig. 7BCJ).

To further verify the relationship between COMMD2/3/10 and immune infiltration in HCC, we used the TIMER database to evaluate the correlations of these COMMD family members with tumor purity and the levels of infiltrating immune cells. The results revealed that the mRNA levels of COMMD2/3/10 were correlated with HCC tumor purity and immune infiltration levels in HCC (*p* < 0.05) (Fig. 7K, L, M). The expression of COMMD2/3/10 showed a significant positive correlation with the infiltration of B cells (r = 0.389, *p* = 7.13e− 14; r = 0.231, *p* = 1.49e− 5 and r = 0.178, *p* = 9.30e− 4, respectively), CD8+ T cells (r = 0.304, *p* = 9.28e− 09; r = 0.142, *p* = 8.57e− 3 and r = 0.107, *p* = 4.80e− 2, respectively), CD4+ T cells (r = 0.405, *p* = 5.32e− 15; r = 0.239, *p* = 7.29e− 6 and r = 0.198, *p* = 2.19e− 4, respectively), macrophages (r = 0.495, *p* = 1.73e− 22; r = 0.319, *p* = 1.60e− 9 and r = 0.227, *p* = 2.36e− 5, respectively), neutrophils (r = 0.465, *p* = 5.92e− 20; r = 0.189, *p* = 4.25e− 4 and r = 0.354, *p* = 1.30e− 11, respectively) and DCs (r = 0.45, *p* = 2.35e− 18; r = 0.258, *p* = 1.40e− 6 and r = 0.193, *p* = 3.34e− 4, respectively) (*p* < 0.05).

The TIMER database was also used to explore the correlations between COMMD2/3/10 expression and immune cell markers (Supplementary Table 3). After adjusting for purity, COMMD2/3/10 expression was positively correlated with the majority of gene markers in different functional T cells (CD8+ T, T helper 1 (Th1), Th2, etc.), B cells, DCs, neutrophils, and NKs, which was consistent with the results in Fig. 7. In particular, COMMD2 expression was almost significantly correlated with all markers of immune cells; specifically, COMMD2 expression was closely correlated (|Correlation coefficient| > 0.4) with CD8+ T cells (CD45 (PTPRC)), M1 macrophages (IRF5), neutrophils (IRF5), DCs (IRF5), Th1 cells (IRF5), and regulatory T cells (Tregs) (CCR8, STAT5B) in HCC.

COMMD3 had a strong correlation with markers of monocytes, tumor-associated macrophages (TAMs), M1 macrophages, neutrophils and Tregs in HCC. COMMD3 was closely correlated (|Correlation coefficient| > 0.25) with monocytes (CD14), M1 macrophages (IRF5), DCs (BDCA-4 (NRP1)), Th1 cells (STAT1), Th2 cells (STAT6), and Tregs (STAT5B).

COMMD10 has a strong association with markers of M1 macrophages, neutrophils, NKs, dendritic cells, and subsets of T cells (including Th1 cells, Th2 cells, Tfh cells, Th17 cells, and Tregs). These results strongly suggested that COMMD2/3/10 expression correlates with the infiltration of immune cells in HCC.

### Genetic mutations of COMMD proteins and their associations with OS, DFS, and PFS in HCC patients

**Fig. 8:**
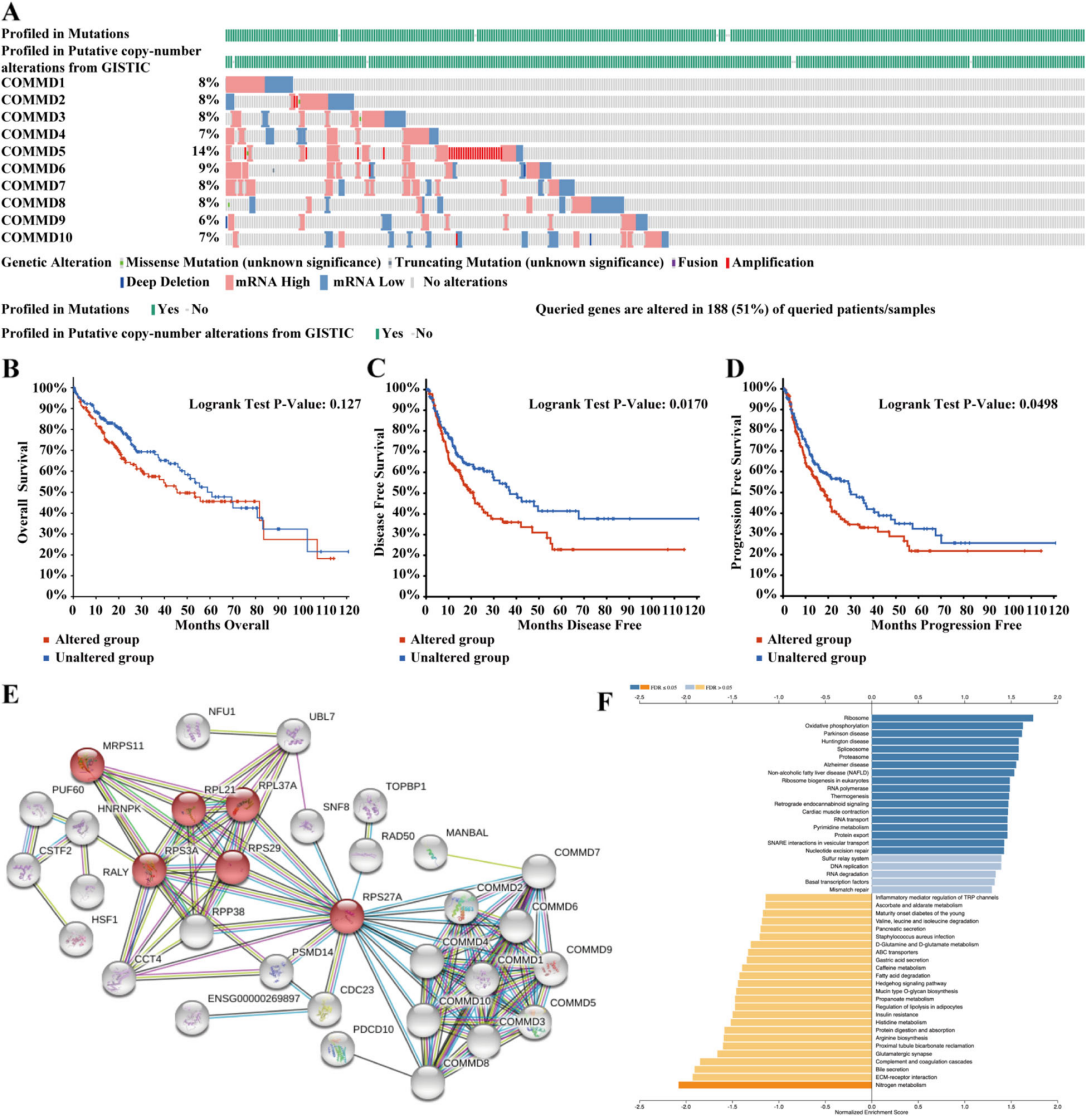
Genetic mutations, network, and GSEA of COMMD proteins. **A** Genetic mutation analysis of COMMD proteins (cBioPortal). **B** Relationship between genetic mutations in COMMD proteins and OS. **C** Relationship between genetic mutations in COMMD proteins and DFS. **D** Relationship between genetic mutations in COMMD proteins and PFS. **E** Network comprising COMMD1–10 and their most closely associated genes (STRING). **F** GSEA of the COMMD protein family members

As indicated in Fig. 8A, a high mutation rate of COMMD proteins was observed in HCC patients. COMMD proteins were altered in 188 samples from 369 HCC patients (51%); COMMD5 had the highest mutation rate (14%). The results from the K-M plot and log-rank test showed that genetic alterations of COMMD proteins were not significantly associated with OS (Fig. 8B, *p* = 0.127), but were associated with shorter DFS (Fig. 8C, *p* = 0.0170) and PFS (Fig. 8D, *p* = 0.0498) in HCC patients. These results implied that genetic alterations of COMMD proteins could significantly affect HCC patient prognosis.

### Predicted functions and pathways affected by the changes in COMMD and closely associated genes in HCC patients

We used GEPIA2 to detect the five most closely genes for each COMMD family member. The STRING database was then applied to construct a network for comprising COMMD and the 50 identified genes (Fig. 8E). The results showed that the most enriched Kyoto Encyclopedia of Genes and Genomes (KEGG) pathway was the ribosome, and ribosome-related genes, including RPS27A, RPS29, RPL37A, RPS3A, RPL21 and MRPS11, were closely associated with the COMMD genes (Fig. 8E). RPS27A may be the key node for the COMMD proteins in HCC. Finally, the LinkedOmics database was used for GSEA of the COMMD proteins to investigate potential biological processes and pathways. In accordance with the STRING results, high expression of COMMD proteins may be related to the ribosome (Fig. 8F).

### Validation of COMMD3 mRNA expression as an independent prognostic factor in GSE14520 dataset and HCC patients

**Fig. 9:**
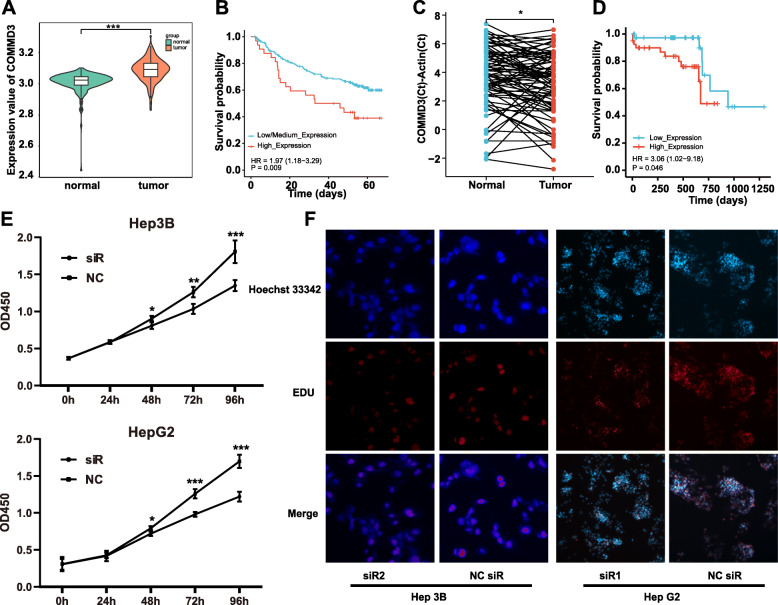
The expression and prognostic value of COMMD3 was validated in GSE14520 and HCC patients. **A** COMMD3 expression in GSE14520 datasets between one thirds of the highest expression and two thirds of the lowest expression. **B** K-M OS curves in GSE14520 datasets stratified by COMMD3 expression. **C** COMMD3 expression in normal and tumor tissue of 80 HCC patients. **D** K–M OS curves in 80 HCC patients stratified by COMMD3 expression. **E** CCK-8 cell proliferation assay results in Hep3B and HepG2 cells transfected with si-COMMD3, or negative control (NC). **F** EdU cell proliferation assay results in Hep3B and HepG2 cells transfected with si-COMMD3, or NC. * *p* < 0.05, *** *p* < 0.001

As shown in Fig. 9A, COMMD3 expression was higher in tumor tissues than normal tissues in GSE14520 dataset between one thirds of the highest expression and two thirds of the lowest expression (*p* < 0.001). The K-M curve and log-rank test analysis indicated that the mRNA levels of COMMD3 were significantly associated with OS (Fig. 9B). QT-PCR results showed that the subtracts mean C_T_ of COMMD3 and Actin were higher in normal tissue than tumor tissue using paired t-test, which mean COMMD3 were more highly expressed in tumor tissues than in paired normal tissues from 80 HCC patients (*p* < 0.05) (Fig. 9C). Higher COMMD3 expression indicated shorter OS (*p* < 0.05) (Fig. 9D).

### COMMD3 siRNA influence in human HCC cells proliferation

Cell proliferation activity was examined in Hep3B and HepG2 cells using CCK-8 and EdU assays. As shown in Fig. 9E, si-COMMD3 significantly decreased the OD value at 48 h, 72 h and 96 h in both Hep3B (up) and HepG2 (below) cell lines (*p* < 0.05). The EdU results (Fig. 9F) showed that si-COMMD3 could inhibit the expression ratio of EdU in the two cell lines. The present results demonstrated that si-COMMD3 suppressed the proliferation of human HCC cells.

## Discussion

Although the function of COMMD7 has been partially confirmed in the tumorigenesis and prognosis of HCC [12], the function of other COMMD family members in HCC has not been explored. The present study is the first to report the mRNA expression, prognostic value, and immune infiltration of the all COMMD family members in cancer. A high mutation rate (51%) of COMMD proteins was observed in HCC patients, and genetic alterations in COMMD proteins were associated with shorter DFS and PFS. A network was constructed for the COMMD genes and their 50 most closely related genes. The most enriched KEGG pathway was the ribosome, which was in agreement with the GSEA results. In addition, the prognostic value of COMMD proteins in advanced HCC has been identified. The expression and survival value of COMMD3 were validated in GSE14520 and 80 HCC patients, and the COMMD3 functional studies were performed in of Human HCC cell lines. Our research contributes to the field by advancing available knowledge, improving treatment design, and enhancing prognostic accuracy for HCC patients.

Among the COMMD family members, COMMD1 is the best characterized. Several studies have reported that COMMD1 inhibits cancer progression through anti-inflammatory activities. COMMD1 is an important negative regulator of NF-κB, while activation of canonical NF-κB signaling increases prostate cancer cell survival [29]. Besides, Li et al. showed that COMMD1 repress inflammation-related genes to protect mice from colitis-associated cancer [30]. In neuroblastoma cells, COMMD1 can form a complex with other proteins to inhibit cyclin D1 expression, G1/S transition, and proliferation. Consistent with these studies, the current study showed that COMMD1 is a tumor suppressor gene in HCC. The TCGA dataset revealed that COMMD1 expression was higher in HCC tissues than in normal tissues, and the mRNA expression of COMMD1 was significantly associated with patients’ individual cancer stage and tumor grade. Moreover, higher mRNA levels of COMMD1 significantly corresponded to longer OS in HCC patients.

To date, the expression or function of COMMD2 in human cancer remains unknown. Under normal physiological conditions, COMMD2 interacts with the epithelial sodium channel (ENaC) to control Na + homeostasis, extracellular fluid volume, and blood pressure [31]. This study showed that COMMD2 expression was higher in HCC tissues than in normal tissues and was correlated with tumor grade and individual cancer stage. However, high COMMD2 expression was also associated with poor prognosis in all patients with HCC. In addition, we found that COMMD2 showed a significant positive correlation to the infiltration of CD8+ T cells, CD4+ T cells, macrophages and DCs in HCC, suggesting that COMMD2 contributes to the recruitment and regulation of immune infiltrating cells to influence prognosis in HCC. COMMD2 expression mainly correlates with the expression of markers of different subsets of Th cells, including Th1 cells (IRF5) and Tregs (CCR8, STAT5B). This suggests a role for COMMD2 in regulating tumor infiltration of Th cells. As Tregs play an important role in tumor immunosuppression, this may partly explain why COMMD2 is an oncogene. Taken together, our findings indicate that COMMD2 plays an important role in regulating the tumor infiltration of immune cells in HCC.

High expression of COMMD3 was found in prostate cancer, and could promote tumor cell migration/invasiveness that associated with tumor recurrence and poor survival [32]. Similarly, high COMMD3 expression was found in HCC tissue in this study and was an independent prognostic factor for longer OS. A lower mRNA level of COMMD3 was significantly associated with favorable OS in all HCC patients. The results from human HCC cells lines supported that lower expression of COMMD3 may influence tumor development via suppressing cell proliferation. In addition, the mRNA expression of COMMD3 was associated with all immune cells and some immune cell markers, including CD14 (monocytes), IRF5 (M1 macrophages), BDCA-4 (NRP1) (DCs), STAT1 (Th1 cells), STAT6 (Th2 cells), and STAT5B (Tregs), in HCC. Marderstein et al. revealed that COMMD3 gene expression correlates with a Th cell phenotype in human tissues [33]. Consistent with this, COMMD3 expression has a strong correlation with Th1 (STAT1) cells, Th2 (STAT6) cells, and Tregs (STAT5B) in this study. We also observed that COMMD3 expression was strongly correlated with M1 macrophages. One reason for this is COMMD3 could stimulate interferon expression to influence immunity [34], while macrophages can strengthen their killing effect on tumor cells by acting interferon. These results indicated that COMMD3 could be used as a novel prognostic biomarker in HCC.

COMMD4 maintains genomic stability via regulating chromatin remodel at sites of DNA double-strand breaks to support cell survival [35]. Higher expression of COMMD4 has been found in non-small cell lung cancer (NSCLC), and was associated with poor prognosis in adenocarcinoma (ADC) [36]. The depletion of COMMD4 markedly reduced cell proliferation and enhanced cell death after exposure to DNA damaging agents [36]. However, in our study, lower expression of COMMD4 related to poor prognosis. Since liver is an important detoxification organ of the human body, tumor or normal cells always encounter toxin leading to DNA injury. Therefore, in HCC cells, higher expression of COMMD4 may mean stronger genomic stability for tumor cell proliferation and stronger toxic adaptive capacity. While in other organs with less toxic, higher expression of COMMD4 means that tumor cells have already accumulated more toxic substances. Thus, it’s different from other tumors that higher COMMD4 levels indicate favorable OS in HCC patients.

COMMD5 is crucial for the cytoskeletal stability, and silencing COMMD5 leads to major reorganization of the actin and microtubule networks. According to a report by Campion, COMMD5 participates in long-range endosome transport, provides the strength to deform, and helps in the fusion of vesicles to sorting endosomes [37]. Their research also indicated that lower COMMD5 expression was found in renal carcinoma and COMMD5 suppress cancer development via cell growth, migration, and differentiation [38]. In this study, we found that COMMD5 are higher expression in HCC tissues than in normal tissues. In patients with grade 3 HCC, high COMMD5 expression was related to poor OS and was an independent prognostic factor for shorter OS, but among all HCC patients, it showed no significant value. The rate of genetic mutation was highest in 10 COMMD proteins and high genetic mutation of COMMD5 may lead to cell death via destroying stability of the cytoskeleton. These results indicated that COMMD5 may play a different role in the tumorigenesis and malignant progression of HCC.

COMMD6 is the most well-studied member of the COMMD proteins in human cancer. The mRNA expression of COMMD6 is higher in 20 types of human cancer, such as HCC, colorectal cancer (CRC), and low-grade glioma than in their corresponding healthy tissues [39]. Eleven types of cancer, including adrenocortical carcinoma, pheochromocytoma and paraganglioma and ovarian cancer, showed lower levels of COMMD6 expression than their healthy counterparts. High COMMD6 expression is associated with shorter OS and DFS in patients with head and neck squamous cell carcinoma, cholangiocarcinoma and adrenocortical carcinoma, but is associated with longer OS and DFS in patients with low-grade glioma and uveal melanoma [39]. In the present study, although COMMD6 expression was higher in HCC tissues than in normal tissues, its association with OS was not statistically significant. Further investigations are needed to explore the oncogenic role of COMMD6 in HCC.

Unlike other COMMD family members, COMMD7 is the only member to have been studied with respect to HCC. You et al. [12] revealed that COMMD7 overexpression significantly promotes the migration and invasion of HCC cells by inducing CXCL10 expression. Zheng et al. reported that the expression levels of COMMD7 are higher in HCC tissues and HCC stem cells (HCSCs), and silencing COMMD7 inhibited cell proliferation, migration, and invasion via suppression of NF-κB p65 [13]. In addition, they also found that HCC cell apoptosis was increased when COMMD7 expression was knocked down [40], and higher COMMD7 expression was associated with a significantly poorer prognosis [41]. Consistent with these studies, we showed that COMMD7 expression was higher in HCC tissues than in normal tissues and that higher expression of COMMD7 was related to shorter OS in all HCC patients and those with grade 3 disease. In addition, COMMD7 was strongly associated with the patients’ individual cancer stage and tumor grade.

The stability of COMMD8 depends on COMMD3, and the COMMD3/8 complex functions as an adaptor that can selectively recruit a specific G protein-coupled receptor kinase (GRK) to chemoattractant receptors and promote lymphocyte chemotaxis [42]. Deficiencies in COMMD8 expression impaired B cell migration and humoral immune responses. Elevated COMMD8 expression has been reported to contribute to the progression of HCC cells [43] and the proliferation and migration of NSCLC cells [44]. Similarly, the present study showed that COMMD8 expression was upregulated in HCC tissues and that high COMMD8 expression indicated unfavorable OS in all HCC patients and those with grade 3 disease.

A report by Zhan et al. demonstrated that COMMD9 expression is upregulated in various NSCLC cell lines and tissue samples [45]. Knocking down COMMD9 inhibited proliferation and migration, arrested the cell cycle at the G1/S transition, and induced autophagy in NSCLC cells. Consistent with these studies, we revealed that COMMD9 expression was more highly expressed in HCC tissues than in normal tissues and that high COMMD9 expression indicated poor OS in all HCC patients; furthermore, in patients with grade 3 HCC, high COMMD9 expression was associated with unfavorable OS and was an independent factor for shorter OS.

Similar to COMMD1, COMMD10 plays a role in cancer by targeting the NF-κB pathway. In CRC, COMMD10 reduces p65 nuclear translocation to block NF-κB pathway activation and suppress CRC invasion and metastasis [46]. In the present study, COMMD10 was more highly expressed in HCC tissues than in normal tissues and was associated with the patients’ individual cancer stage and tumor grade. In all HCC patients, high COMMD10 expression was related to shorter OS, but the relationship was not significant. However, in patients with grade 3 HCC, high expression of COMMD10 indicated poor OS. Consistent with the report that COMMD10 is involved in memory T cell differentiation in healthy individuals [47], we found that COMMD10 has a strong association with markers of T cell subsets, including Th1 cells, Th2 cells, Tfh cells, Th17 cells and Tregs, in HCC. Our results suggest that COMMD10 improves HCC patient prognosis via immune infiltration.

## Conclusion

The current study showed that aberrant expression of 10 COMMD family members was associated with clinical cancer stage and pathological tumor grade in HCC patients. In all HCC patients, higher mRNA expression of COMMD1/4 was significantly associated with favorable OS, while higher mRNA expression of COMMD2/3/7/8/9 was associated with poor OS. Multivariate analysis indicated that high mRNA expression of COMMD3 was an independent prognostic factor for shorter OS in all HCC patients. However, in patients with grade 3 HCC, higher COMMD4/10 expression was associated with better OS, whereas higher COMMD3/5/7/8/9 expression was related to poorer OS. Moreover, a high mutation rate of COMMD proteins (51%) was observed in HCC patients, and genetic alterations in COMMD proteins were associated with shorter DFS and PFS. COMMD2/3/10 were associated with tumor-induced immune response activation and immune infiltration in HCC. Then, we showed that COMMD3 was more highly expressed in tumor tissues than in normal tissues from GSE14520 and 80 patients, was associated with poor OS and was an independent prognostic factor for shorter OS. Finally, we validated that knockdown of COMMD3 inhibits human HCC cell lines proliferation in vitro*.* These results indicated that COMMD3 may be a prognostic biomarker for the survival of all HCC patients.

## Supplementary Information

**Additional file 1: Fig. S1** SiRNA efficiency of COMMD3 in Hep 3B and Hep G2 cell lines via Western blotting.

**Additional file 2: Table S1**. The clinical characteristics of 344 HCC patients in TCGA. **Table S2**. The clinical characteristics of 124 patients with grade III HCC in TCGA. **Table S3**. Correlation analysis between COMMD2/3/10 and relate genes and markers of immune cells in TIMER.

## Data Availability

The datasets used and/or analyzed during the current study are available from the corresponding author upon reasonable request.

## Abbreviations

HCC: Hepatocellular carcinoma
OS: Overall survival
COMMD: Copper metabolism MURR1 domain
CXCL10: C-X-C motif chemokine 10
K-M: Kaplan-Meier
TCGA: The Cancer Genome Atlas
DFS: Disease-free survival
PFS: Progression-free survival
ESTIMATE: Estimation of STromal and Immune cells in MAlignant Tumor Tissues using Expression data
TIMER: Tumor immune estimation resource
GEPIA: The Gene Expression Profiling Interactive Analysis
NKs: Natural killer cells
DCs: Dendritic cells
GEPIA2: The Gene Expression Profiling Interactive Analysis 2
GSEA: Gene Set Enrichment Analysis
OD: Optical density
EdU: Cell-Light 5-ethynyl-2-deoxyuridine
Th1: T helper 1
Tregs: Regulatory T cells
TAMs: Tumor-associated macrophages
KEGG: Kyoto Encyclopedia of Genes and Genomes
NSCLC: Non-small cell lung cancer
ADC: Adenocarcinoma
CRC: Colorectal cancer
HCSCs: HCC stem cells
GRK: G protein-coupled receptor kinase
