# Supplementary figures and images for: Transcriptional analysis of the expression, prognostic value and immune infiltration activities of the COMMD protein family in hepatocellular carcinoma
Source: BMC Cancer. 2021 Sep 7;21:1001. doi: 10.1186/s12885-021-08699-3 (PMC8424899; doi:10.1186/s12885-021-08699-3)

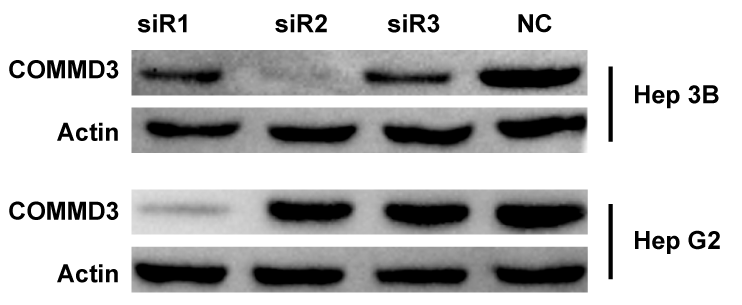


Fig S1 SiRNA efficiency of COMMD3 in Hep 3B and Hep G2 cell lines via Western blotting.

Supplement: Supplementary file 1 — Additional file 1: Fig. S1 SiRNA efficiency of COMMD3 in Hep 3B and Hep G2 cell lines via Western blotting. [file 12885_2021_8699_MOESM1_ESM.docx]
