# Supplementary material for: Transcriptional analysis of the expression, prognostic value and immune infiltration activities of the COMMD protein family in hepatocellular carcinoma
Source: BMC Cancer. 2021 Sep 7;21:1001. doi: 10.1186/s12885-021-08699-3 (PMC8424899; doi:10.1186/s12885-021-08699-3)
Supplement: Supplementary file 2 — Additional file 2: Table S1. The clinical characteristics of 344 HCC patients in TCGA. Table S2. The clinical characteristics of 124 patients with grade III HCC in TCGA. Table S3. Correlation analysis between COMMD2/3/10 and relate genes and markers of immune cells in TIMER. [file 12885_2021_8699_MOESM2_ESM.docx]

Supplemental Table 1. The clinical characteristics of 344 HCC patients in TCGA

| Characteristic | Freq |
| --- | --- |
| Gender, No. (%) |  |
| Male | 234 (68%) |
| Female | 110 (32%) |
| Histological_Grade, No. (%) | |
| G1+G2 | 214 (62%) |
| G3+G4 | 130 (38%) |
| age, No. (%) |  |
| >60 | 173 (50%) |
| ≤60 | 171 (50%) |
| Tumor_Stage, No. (%) | |
| Stage1+2 | 254 (74%) |
| Stage3+4 | 90 (26%) |
| COMMD1, No. (%) |  |
| Low_expression | 206 (60%) |
| High_expression | 138 (40%) |
| COMMD2, No. (%) |  |
| Low_expression | 194 (56%) |
| High_expression | 150 (44%) |
| COMMD3, No. (%) |  |
| Low_expression | 205 (60%) |
| High_expression | 139 (40%) |
| COMMD4, No. (%) |  |
| Low_expression | 200 (58%) |
| High_expression | 144 (42%) |
| COMMD5, No. (%) |  |
| Low_expression | 203 (59%) |
| High_expression | 141 (41%) |
| COMMD6, No. (%) |  |
| Low_expression | 218 (63%) |
| High_expression | 126 (37%) |
| COMMD7, No. (%) |  |
| Low_expression | 204 (59%) |
| High_expression | 140 (41%) |
| COMMD8, No. (%) |  |
| Low_expression | 216 (63%) |
| High_expression | 128 (37%) |
| COMMD9, No. (%) |  |
| Low_expression | 233 (68%) |
| High_expression | 111 (32%) |
| COMMD10, No. (%) |  |
| Low_expression | 201 (58%) |
| High_expression | 143 (42%) |

Supplemental Table 2. The clinical characteristics of 124 patients with grade III HCC in TCGA

| Characteristic | Freq |
| --- | --- |
| gender, No. (%) | |
| Male | 81 (65%) |
| Female | 43 (35%) |
| age, No. (%) | |
| >60 | 54 (44%) |
| ≤60 | 70 (56%) |
| stage_event_pathologic_stage, No. (%) | |
| Stage1+2 | 90 (73%) |
| Stage3+4 | 34 (27%) |
| COMMD1, No. (%) | |
| Low_expression | 72 (58%) |
| High_expression | 52 (42%) |
| COMMD2, No. (%) | |
| Low_expression | 72 (58%) |
| High_expression | 52 (42%) |
| COMMD3, No. (%) | |
| Low_expression | 75 (60%) |
| High_expression | 49 (40%) |
| COMMD4, No. (%) | |
| Low_expression | 73 (59%) |
| High_expression | 51 (41%) |
| COMMD5, No. (%) | |
| Low_expression | 72 (58%) |
| High_expression | 52 (42%) |
| COMMD6, No. (%) | |
| Low_expression | 75 (60%) |
| High_expression | 49 (40%) |
| COMMD7, No. (%) | |
| Low_expression | 68 (55%) |
| High_expression | 56 (45%) |
| COMMD8, No. (%) | |
| Low_expression | 77 (62%) |
| High_expression | 47 (38%) |
| COMMD9, No. (%) | |
| Low_expression | 69 (56%) |
| High_expression | 55 (44%) |
| COMMD10, No. (%) | |
| Low_expression | 77 (62%) |
| High_expression | 47 (38%) |

Supplementary Table 3. Correlation analysis between COMMD2/3/10 and relate genes and markers of immune cells in TIMER

| Description | Gene markers | COMMD2 | | | | COMMD3 | | | | COMMD10 | | | |
| --- | --- | --- | --- | --- | --- | --- | --- | --- | --- | --- | --- | --- | --- |
|  |  | None | | Purity | | None | | Purity | | None | | Purity | |
|  |  | Cor | P | Cor | P | Cor | P | Cor | P | Cor | P | Cor | P |
| CD8+ | CD8A | 0.17 | ** | 0.17 | ** | 0.07 | 0.19 | 0.06 | 0.27 | 0.04 | 0.48 | 0.02 | 0.66 |
|  | CD8B | 0.09 | 0.08 | 0.10 | 0.05 | 0.06 | 0.23 | 0.06 | 0.26 | -0.01 | 0.82 | -0.02 | 0.75 |
|  | CD45 (PTPRC) | 0.41 | *** | 0.43 | *** | 0.12 | * | 0.12 | * | 0.30 | *** | 0.29 | *** |
| T cell | CD3D | 0.13 | ** | 0.15 | ** | 0.08 | 0.12 | 0.08 | 0.15 | -0.05 | 0.38 | -0.05 | 0.40 |
|  | CD3E | 0.14 | ** | 0.15 | ** | 0.02 | 0.71 | 0.01 | 0.89 | 0.01 | 0.91 | 0.00 | 0.98 |
|  | CD2 | 0.13 | ** | 0.15 | ** | 0.04 | 0.48 | 0.03 | 0.57 | -0.03 | 0.59 | -0.03 | 0.56 |
| B cell | CD19 | 0.20 | *** | 0.19 | *** | 0.11 | * | 0.09 | 0.10 | 0.02 | 0.66 | 0.01 | 0.83 |
|  | CD79A | 0.12 | * | 0.13 | * | 0.00 | 0.97 | -0.02 | 0.76 | -0.05 | 0.32 | -0.07 | 0.21 |
|  | CD27 | 0.15 | ** | 0.16 | ** | 0.08 | 0.15 | 0.08 | 0.16 | -0.03 | 0.59 | -0.04 | 0.50 |
|  | CD20 (KRT20) | 0.18 | *** | 0.21 | *** | 0.16 | ** | 0.14 | ** | 0.20 | *** | 0.21 | *** |
| monocyte | CD14 | -0.29 | *** | -0.29 | *** | -0.26 | *** | -0.25 | *** | -0.12 | * | -0.11 | * |
|  | CD115 (CSF1R) | 0.20 | *** | 0.21 | *** | 0.13 | * | 0.12 | * | 0.11 | * | 0.10 | 0.07 |
| TAM | CCL2 | 0.17 | *** | 0.17 | ** | 0.08 | 0.12 | 0.06 | 0.27 | 0.05 | 0.33 | 0.04 | 0.42 |
|  | CD68 | 0.21 | *** | 0.20 | *** | 0.12 | * | 0.11 | 0.05 | 0.10 | 0.06 | 0.08 | 0.14 |
|  | IL10 | 0.24 | *** | 0.23 | *** | 0.14 | ** | 0.13 | * | 0.15 | ** | 0.13 | * |
| M1 macrophage | INOS (NOS2) | 0.16 | ** | 0.15 | ** | 0.07 | 0.20 | 0.06 | 0.28 | 0.16 | ** | 0.16 | ** |
|  | CD80 | 0.35 | *** | 0.35 | *** | 0.17 | *** | 0.18 | ** | 0.23 | *** | 0.21 | *** |
|  | IRF5 | 0.45 | *** | 0.44 | *** | 0.33 | *** | 0.34 | *** | 0.29 | *** | 0.29 | *** |
|  | IL6 | 0.08 | 0.11 | 0.09 | 0.11 | -0.08 | 0.13 | -0.09 | 0.09 | 0.01 | 0.85 | 0.00 | 0.99 |
|  | CD64 (FCGR1A) | 0.21 | *** | 0.22 | *** | 0.15 | ** | 0.15 | ** | 0.07 | 0.18 | 0.05 | 0.32 |
| M2 Macrophage | CD163 | 0.18 | *** | 0.18 | *** | 0.05 | 0.36 | 0.04 | 0.45 | 0.16 | ** | 0.15 | ** |
|  | CD206 (MRC1) | 0.06 | 0.26 | 0.07 | 0.22 | -0.05 | 0.34 | -0.06 | 0.31 | 0.09 | 0.10 | 0.07 | 0.18 |
|  | VSIG4 | 0.16 | ** | 0.16 | ** | 0.11 | * | 0.11 | * | 0.11 | * | 0.09 | 0.08 |
|  | MS4A4A | 0.15 | ** | 0.16 | ** | 0.09 | 0.09 | 0.08 | 0.13 | 0.15 | ** | 0.14 | * |
| Neutrophils | CD66 (CEACAM8) | 0.06 | 0.24 | 0.07 | 0.22 | 0.06 | 0.22 | 0.07 | 0.17 | 0.07 | 0.20 | 0.08 | 0.16 |
|  | CD11b (ITGAM) | 0.30 | *** | 0.31 | *** | 0.17 | ** | 0.17 | ** | 0.24 | *** | 0.24 | *** |
|  | CD15 (FUT4) | 0.43 | *** | 0.43 | *** | 0.23 | *** | 0.19 | *** | 0.18 | *** | 0.17 | ** |
| Natural Killer cell | KIR2DL1 | 0.03 | 0.53 | 0.00 | 0.94 | -0.02 | 0.69 | -0.05 | 0.35 | 0.02 | 0.77 | 0.02 | 0.73 |
|  | KIR2DL3 | 0.17 | ** | 0.18 | *** | 0.16 | ** | 0.15 | ** | 0.12 | * | 0.11 | * |
|  | KIR3DL1 | 0.09 | 0.08 | 0.09 | 0.08 | 0.03 | 0.51 | 0.01 | 0.80 | 0.13 | * | 0.14 | ** |
|  | KIR3DL2 | 0.10 | 0.06 | 0.11 | * | 0.00 | 1.00 | 0.01 | 0.84 | 0.03 | 0.60 | 0.04 | 0.51 |
|  | CD65 (NCAM1) | 0.26 | *** | 0.27 | *** | 0.13 | * | 0.12 | * | 0.17 | *** | 0.18 | *** |
|  | CD335 (NCR1) | 0.15 | ** | 0.17 | ** | -0.03 | 0.50 | -0.02 | 0.72 | 0.19 | *** | 0.20 | *** |
| Dendritic cell | BDCA-1 (CD1C) | 0.24 | *** | 0.24 | *** | 0.08 | 0.12 | 0.07 | 0.21 | 0.04 | 0.42 | 0.05 | 0.40 |
|  | CD141 (THBD) | 0.13 | * | 0.12 | * | -0.05 | 0.33 | -0.07 | 0.18 | 0.08 | 0.11 | 0.06 | 0.26 |
|  | BDCA-4 (NRP1) | 0.48 | *** | 0.47 | *** | 0.32 | *** | 0.29 | *** | 0.31 | *** | 0.30 | *** |
|  | CD123 (IL3RA) | -0.02 | 0.74 | -0.02 | 0.77 | 0.00 | 1.00 | -0.03 | 0.63 | -0.10 | * | -0.10 | 0.06 |
|  | CD11c (ITGAX) | 0.31 | *** | 0.31 | *** | 0.14 | ** | 0.14 | * | 0.19 | *** | 0.19 | *** |
| Th1 | T-bet (TBX21) | 0.12 | * | 0.13 | * | -0.02 | 0.66 | -0.04 | 0.47 | 0.03 | 0.50 | 0.03 | 0.56 |
|  | STAT4 | 0.22 | *** | 0.24 | *** | 0.08 | 0.14 | 0.06 | 0.23 | 0.06 | 0.22 | 0.07 | 0.19 |
|  | STAT1 | 0.48 | *** | 0.49 | *** | 0.24 | *** | 0.23 | *** | 0.27 | *** | 0.27 | *** |
| Th2 | GATA3 | 0.21 | *** | 0.23 | *** | 0.02 | 0.69 | 0.01 | 0.89 | 0.07 | 0.19 | 0.07 | 0.22 |
|  | STAT6 | 0.32 | *** | 0.31 | *** | 0.25 | *** | 0.22 | *** | 0.25 | *** | 0.22 | *** |
|  | IL13 | 0.10 | 0.07 | 0.09 | 0.08 | 0.01 | 0.87 | -0.01 | 0.90 | 0.16 | ** | 0.15 | ** |
| Tfh | BCL6 | 0.37 | *** | 0.37 | *** | 0.18 | *** | 0.18 | *** | 0.30 | *** | 0.31 | *** |
|  | IL21 | 0.09 | 0.10 | 0.10 | 0.06 | 0.04 | 0.40 | 0.05 | 0.37 | 0.07 | 0.15 | 0.09 | 0.09 |
| Th17 | STAT3 | 0.33 | *** | 0.34 | *** | 0.08 | 0.14 | 0.07 | 0.18 | 0.31 | *** | 0.29 | *** |
|  | IL17A | 0.11 | * | 0.12 | * | 0.05 | 0.36 | 0.04 | 0.41 | 0.13 | * | 0.14 | ** |
|  | RORgt (RORC) | 0.02 | 0.65 | 0.02 | 0.72 | -0.03 | 0.54 | -0.02 | 0.67 | 0.15 | ** | 0.16 | ** |
| Treg | FOXP3 | 0.29 | *** | 0.30 | *** | 0.15 | ** | 0.16 | ** | 0.21 | *** | 0.20 | *** |
|  | CD25 (IL2RA) | 0.24 | *** | 0.24 | *** | 0.10 | 0.06 | 0.09 | 0.11 | 0.14 | ** | 0.12 | * |
|  | CCR8 | 0.43 | *** | 0.45 | *** | 0.15 | ** | 0.14 | ** | 0.29 | *** | 0.28 | *** |
|  | STAT5B | 0.55 | *** | 0.56 | *** | 0.25 | *** | 0.25 | *** | 0.49 | *** | 0.49 | *** |
| T cell exhaustion | PD-1 (PDCD1) | 0.19 | *** | 0.18 | *** | 0.10 | * | 0.09 | 0.09 | 0.03 | 0.53 | 0.01 | 0.90 |
|  | CTLA4 | 0.18 | *** | 0.19 | *** | 0.13 | * | 0.12 | * | 0.05 | 0.30 | 0.05 | 0.33 |
|  | LAG3 | 0.16 | ** | 0.15 | ** | 0.09 | 0.08 | 0.09 | 0.11 | -0.01 | 0.84 | -0.02 | 0.74 |
|  | TIM-3 (HAVCR2) | 0.27 | *** | 0.28 | *** | 0.14 | ** | 0.14 | ** | 0.15 | ** | 0.13 | * |
|  | GZMB | 0.04 | 0.49 | 0.03 | 0.64 | 0.06 | 0.21 | 0.04 | 0.42 | 0.02 | 0.67 | 0.02 | 0.76 |

None, correlation without adjustment; Purity, correlation adjusted by purity; TAM, tumor-associated macrophage; Th, T helper cell; Tfh, Follicular helper T cell; Treg, regulatory T cell; Cor, R value of Spearman’s correlation

* P<0.05; ** P<0.01; *** P<0.001
